# The Relationship Between Morphological Awareness and Reading Comprehension Among Chinese Children

**DOI:** 10.3389/fpsyg.2019.00054

**Published:** 2019-02-01

**Authors:** Ruibo Xie, Jie Zhang, Xinchun Wu, Thi Phuong Nguyen

**Affiliations:** ^1^Beijing Key Laboratory of Applied Experimental Psychology, Faculty of Psychology, Beijing Normal University, Beijing, China; ^2^Department of Curriculum and Instruction, University of Houston, Houston, TX, United States; ^3^Collaborative Innovation Center of Assessment toward Basic Education Quality, Beijing Normal University, Beijing, China

**Keywords:** compounding awareness, homograph awareness, homophone awareness, reading comprehension, Chinese

## Abstract

The present study examined the developmental relationship between morphological awareness and reading comprehension, using a 1-year longitudinal study with a sample of 439 Chinese-speaking students in Grades 1, 3, and 5, respectively. Children’s text reading and three components of morphological awareness: homophone awareness, homograph awareness and compounding awareness were measured. After controlling for word reading, vocabulary knowledge, IQ, rapid automatized naming and phonological awareness measured at the initial level, the structural equation modeling results indicated that children’s compounding awareness made a significant direct contribution to reading comprehension only from Grade 5 to 6. Children’s reading comprehension also made a unique contribution to compounding awareness from Grade 5 to 6. Thus a reciprocal relationship between compounding awareness and reading comprehension was found for Grade 5 to 6. Reading comprehension in Grades 3 and 5 predicted homophone awareness in Grades 4 and 6 were marginal significance, respectively, but initial homophone awareness did not predict later reading comprehension across grades. Furthermore, there no unique connection between homograph awareness and reading comprehension across grades. These findings suggest a dynamic relationship between different aspects of morphological awareness and reading comprehension in Chinese-speaking children across elementary school years.

## Introduction

The ability to read and comprehend what they read is necessary skill for children to learn during their elementary school years. One most important skill that contribute to reading development is children’s awareness of morphemes (morphological awareness) in the oral language (Nagy et al., 2003; Shu et al., 2003). Morphological awareness has been shown to be a unique contribution to reading comprehension across languages beyond substantive controls (e.g., Saiegh-Haddad and Geva, 2008; Deacon et al., 2014; Ruan et al., 2018). Morphological awareness plays an important role in reading development in Chinese (Shu et al., 2006; Liu and McBride-Chang, 2010; Cheng et al., 2017) and it involves the integration of semantic, phonological, and syntactic information (Hanley, 2005; Li et al., 2017). Extensive research has shown that morphological awareness is a stronger correlate of reading comprehension than phonological awareness in Chinese, due to the morphosyllabic nature of Chinese (see a meta-analysis, in Ruan et al., 2018). In addition, Kuo and Anderson (2006) noted that “extensive exposure to print could lead to better morphological awareness” (p. 175). There is increasing evidence that the relationship between morphological awareness and reading comprehension could be reciprocal in Chinese (Wu et al., 2009; Cheng et al., 2017). Few studies have examined this relation in a longitudinal design and previous research only taps one aspect of morphological awareness in Chinese. The current study expands the previous research by investigating three components of morphological awareness in Chinese: compounding awareness, homograph awareness and homophone awareness (Liu and McBride-Chang, 2010; Liu et al., 2013; Cheng et al., 2017; Ruan et al., 2018) and the possible reciprocal relation between reading comprehension and each component of morphological awareness in a 1-year longitudinal design.

Going beyond previous research which focuses on a particular age group, the current research involves a full span of elementary grades 1–6. Previous research suggests the increasing importance of morphological awareness in reading development with grades and a reciprocal relationship in older children (e.g., Kuo and Anderson, 2003, 2006; Wu et al., 2009; Deacon et al., 2014). However, the developmental changes in the longitudinal relation and directionality between the three components of morphological awareness and reading comprehension in Chinese across elementary grades are less understood.

Morpheme is the smallest meaningful or consistently occurring element in a language. Chinese as a morphosyllabic language that uses a logographic script. Most Chinese morphemes only represent a single character or single syllable (Packard, 2000; Shu et al., 2003; Hanley, 2005; Li et al., 2012). As the smallest meaningful units in a language, morphemes can be combined to form a word in three ways, including inflection, derivation, and compounds (Kuo and Anderson, 2006). More than 75% of Mandarin Chinese words are compounds, composed of two or more morphemes or characters (Kuo and Anderson, 2006), and very few inflectional and derivational words exist in Chinese. In addition, Mandarin Chinese has been evolving in the direction of making its syllables ever shorter and structurally simpler, which results in a vast number of homophones (e.g., 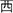/xi1/ “west” and 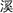 /xi1/ “brook”) and homonyms (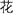/hua1/ “flower”, “spend”) among words (Shu and Anderson, 1997; Li et al., 2017). Research shows that about 70%, or approximately 1,300 Mandarin syllables represent more than one morpheme, and on average one syllable corresponds to five morphemes or characters (Tang, 1995; Shu and Anderson, 1997; Duanmu, 2007).

Morphological awareness refers to the capacity to reflect on and manipulate morphemes and the morphological structure of words (Carlisle, 1995; Kuo and Anderson, 2006). Based on the characteristics of the Chinese language described above, it is commonly accepted that Chinese morphological awareness comprises both morpheme/character and word levels and involves at least three components: homograph awareness, homograph awareness, and compounding awareness (Liu and McBride-Chang, 2010; Liu et al., 2013; Cheng et al., 2017; Ruan et al., 2018). At the morpheme level, morpheme awareness involves the ability to identify and manipulate a specific morpheme, including homograph awareness and homograph awareness (Liu et al., 2013). Due to the prevalence of homophonous morphemes, it is crucial to distinguish among various morphemes that sound the same. Homophone awareness helps children distinguish homophonic characters that share the same syllable (e.g., /ji4/), but with different morphemes (e.g., 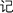
“remember,” 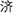
“benefit,” 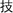
“technique,” 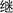
“follow,” 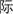
“border,” 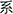
“tie”). Moreover, the same character (e.g., 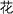/hua1/) can represent different meanings (e.g., 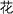/hua1/) means “flower” or “to spend”). Homograph awareness helps children to discern that a single written character may represent different meanings, when present in any given word (e.g., 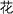/hua1/ in 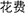/hua1fei4/ means “to spend,” but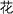/hua1/ in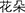/hua1duo3/ means “flower”). At the level of word structure, morphological awareness refers to the ability to identify, analyze, and manipulate the morphological structure rules in a word (Liu et al., 2013). Due to the productivity of compound words in Chinese, word-level morphological awareness mainly refers to the awareness of compound word structure. Chinese lexical compounding awareness, that is, “the understanding of how morphemes can be combined sensibly in the Chinese language” (Liu and McBride-Chang, 2010, p. 63), is a significant contributor to reading comprehension in Chinese children, even after controlling for word reading, vocabulary knowledge, and other variables (Carlisle, 2000; Cheng et al., 2017; Li et al., 2017).

Reading comprehension involves the process of meaning-making, and morphemes are perceive and manipulate during semantic information acquisition (Wang et al., 2006; Li and Wu, 2015). A growing body of research has shown that children’s morphological awareness is a unique contribution to reading comprehension across languages (Nagy et al., 2003; Saiegh-Haddad amd Geva, 2008; Kieffer and Lesaux, 2012), especially in Chinese (Shu et al., 2003, 2006; Liu and McBride-Chang, 2010; Zhang et al., 2014; Cheng et al., 2017; Li et al., 2017). Research has consistently shown that morphological awareness can not only promote decoding of words, but also promote vocabulary development, and have a direct or indirect effect on reading comprehension (Liu and McBride-Chang, 2010; Deacon et al., 2014; Cheng et al., 2015, 2017). Notably, a number of studies that have reported a direct contribution of morphological awareness to Chinese reading comprehension did not include word reading and vocabulary knowledge as control variable. In a cross-sectional study with first-grade students in China, failed to detect a unique connection between homograph awareness and reading comprehension (Yeung et al., 2011), after controlling for both word reading and vocabulary knowledge. A longitudinal sample of Chinese students from grades 1 to 2 were assessed, and the results suggested that initial status and growth rates of compounding awareness made a significant direct contribution to reading comprehension at the end of second grade, after controlling for vocabulary knowledge, IQ and phonological awareness; the relationship between initial status and growth rates of compounding awareness and reading comprehension were fully mediated by word reading (Cheng et al., 2017). While previous studies has shown that morphological awareness is a strong concurrent and longitudinal predictor of reading comprehension in Chinese (e.g., McBride-Chang et al., 2007; Tong et al., 2009; Liu and McBride-Chang, 2010; Yeung et al., 2011; Cheng et al., 2017; Li et al., 2017), the results were not consistent when controlled word reading and vocabulary knowledge.

According to the Simple View of Reading, word decoding and linguistic comprehension are two core skills for reading comprehension (Gough and Tunmer, 1986; Hoover and Gough, 1990). In the early stages of elementary school years, children are heavily dependent on the familiar appearance of words for decoding in beginning readers who are learning to read (Chall, 1983; Ehri, 1995, 2005). One meta-analysis showed that word reading would be more important for younger readers than older readers (García and Cain, 2014). As the grade increases, the unique role of morphological awareness in reading development is may of increasing importance (e.g., Kuo and Anderson, 2003, 2006; Wu et al., 2009; Deacon et al., 2014), which shares overlapping characteristics with phonological awareness, orthographic awareness, syntactic awareness, and semantic knowledge (Carlisle, 1995; Shu et al., 2006; Liu et al., 2013; Li et al., 2017). Deacon et al. (2014) noted this possibility, and notion that “there appears to be partial or complete mediation between morphological awareness and reading comprehension by word reading in middle elementary school, with direct effects only between morphological awareness and reading comprehension by upper elementary school.” It is possible that the unique role of morphological awareness in reading development would be more important for older children in upper elementary school, than younger children in the early and middle of primary school years. Therefore, the current research attempts to fill the gap in examining the unique roles of morphological awareness in reading comprehension across early – middle – upper elementary school years, after controlling for both word reading and vocabulary knowledge.

Most existing studies have used compounding awareness, or a latent variable of morphological awareness in studying the relationship between morphological awareness and reading comprehension in Chinese (e.g., Wu et al., 2009; Cheng et al., 2015, 2017). It remains unknown if different aspects of morphological awareness relate to Chinese reading similarly, at different developmental stages. As mentioned earlier, the different aspects of morphological awareness may differ because they focus on different features of language. Compounding awareness focus on word level, involving the understanding of how morphemes can be combined sensibly. In the present study, a compound production task was used to test students’ compounding awareness, which requires students to produce a novel word in response to an orally presented question (Liu and McBride-Chang, 2010). For example, “What should we call a bird like a frog?”; in a separate example, “What do we call a factory that is specially used to recycle glove?” (Liu and McBride-Chang, 2010). The correct answer for the first example is frog-bird (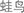/wa1–niao3/), and the correct answer for the second example is glove-recycling-factory (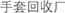/shou3–tao4–hui2–shou1–chang3/). The open-ended task directly taps students’ ability to use both the morphemes and their morphological structure knowledge to produce new and complex words. Thus, compounding awareness could help children to understand the meanings of complex and novel words within texts thereby helping them to understand the meaning of the whole passage.

In addition, homograph awareness and homophone awareness focus on morpheme/character level, involving the ability to differentiate homographs or homophones. In this study, homophone/homographic production task has been used in orally. In homophone production task (Liu and McBride-Chang, 2010), children were first orally presented with a specific morpheme in a two-character word, (e.g., 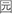/yuan2/ “garden” in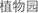/zhi2–wu4–yuan2/ “botanical garden”]; and then asked to produce another words that include this morpheme (e.g., 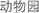/dong4-wu4-yuan2/ “zoo”) and then to produce as many homophones (words containing the same syllable /yuan2/) as possible (e.g., 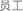/yuan2–gong1/ “employee” or 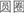/yuan2–quan1/ “ring”). Similarly, in homographic awareness task (Shu et al., 2006), children were first presented with a two-syllable Chinese word (e.g., 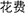/hua1–duo3/ “flower”) that included the target morpheme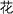/hua1/. Children were asked to produce two words with the target morpheme. One word was supposed to contain the same meaning as the target morpheme (e.g., 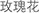/mei2–gui4–hua1/ “rose”) and the other word was supposed to have a different meaning from that of the target morpheme but the same pronunciation and written form, such as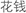/hua1–qian2/ “spend.” Homophone awareness and homograph awareness require sufficient vocabulary knowledge to recognize whether the same syllable represents different orthography/meanings (homophone awareness) and whether the same orthography may represent different meanings (homograph awareness). In order to recognize the specific morpheme, homograph awareness, and homophone awareness may require children are familiar to the vocabulary knowledge, which contains this morpheme. However, children are likely to encounter many morphologically complex words in their reading, they make up at least 40% of all the new words that children encounter in texts; especially the morphologically complex and novel words common in upper elementary school (Nagy and Anderson, 1984; Nagy et al., 1993). Further, Deacon et al. (2014)suggested children’s morphological awareness involved the ability to read key complex and novel words could then support comprehension of texts. Remarkably, beside the characteristic of semantic knowledge of complex and novel words, compounding awareness also shares characteristic with syntactic awareness, given the morphological structure of words is often conditioned by syntactic context (e.g., Li et al., 2017). Thus, there might be a stronger association between children’s compounding awareness and reading comprehension, compared to the association of homograph awareness and homograph awareness with reading comprehension in elementary school years. However, none of the studies have included the three components of morphological awareness (i.e., homograph awareness, homograph awareness and compounding awareness) in the same model simultaneously to explore the unique contributions of each component to reading comprehension across elementary grades.

A reciprocal relationship between morphological awareness and reading comprehension has been proposed and empirically tested. Theoretically, a reciprocal developmental relationship appears to exist between morphological awareness and reading comprehension. Kuo and Anderson (2006) wrote that “extensive exposure to print could lead to better morphological awareness” (p. 175). It is possible that learning to read triggers consciousness of certain morphological features, with increasing exposure to complex print. More specifically, children’s reading experience may promote opportunities for children to develop abstract understanding of critical morphemes and extract the structure of compound words in reading, thus promoting morphological awareness development (Kuo and Anderson, 2006). Empirically, previous studies have discovered a consistent pattern of this reciprocal relationship in English speaking children (Kruk and Bergman, 2013; Deacon et al., 2014), but the findings are mixed in Chinese (Wu et al., 2009; Cheng et al., 2015). In a longitudinal study with English-speaking children, Deacon et al. (2014) found that morphological awareness in Grade 3 predicted growth in reading comprehension at Grade 4, and vice versa. Similarly, Kruk and Bergman (2013) reported a reciprocal relationship: children’s morphological awareness at Grade 1 predicted reading comprehension at Grade 3, and their early reading comprehension also predicted morphological awareness at Grade 3. Currently, there are only two studies focusing on the reciprocal relations between morphological awareness and reading comprehension in Chinese, and the results are inconsistent (Wu et al., 2009; Cheng et al., 2015). Wu et al. (2009) reported an intervention in morphology of characters and words for Chinese children, and the findings showed that morphological awareness had a reciprocal influence on reading comprehension in Grade 3, but only a unidirectional relationship from morphological awareness to reading comprehension was supported in Grade 2. The findings indicated that the reciprocal relationship emerged for older children. In a longitudinal study, Cheng et al. (2015) found that morphological awareness predicted the growth rate of reading comprehension, and the reverse relation was also found from Grades 1 to 2, suggesting a reciprocal developmental relationship between children’s morphological awareness and reading comprehension for Chinese young children.

The inconsistencies among the results of these two studies may be attributable to the difference in variable selection. In Wu et al’s study (2009), children’s morphological awareness in Grade 2 was a latent variable extracted from compounding awareness, homophone awareness and homograph awareness, but in Grade 3, morphological awareness was extracted from compounding awareness and homophone awareness. In Cheng et al. (2015) study, morphological awareness was a latent variable extracted from homograph awareness, homograph awareness and compounding awareness. Moreover, the choice of reading indicator and control variables differs across studies. Cheng et al. (2015) used text reading comprehension and after controlled children’s phonological awareness, IQ and vocabulary knowledge, whereas Wu et al. (2009) used a latent variable, which extracted sentence reading comprehension and vocabulary and/or reading fluency in Grades 2 and 3, and only controlled children’s IQ. The inconsistencies among the results of these studies emphasize the need for further research and motivate the current study.

In addition to word reading and vocabulary knowledge, children’s phonological awareness, rapid automatized naming, and IQ were also found to be associated with their reading comprehension. The phonological awareness have been proved to have a positive impact on reading comprehension. Based on the fact that 80% of Chinese characters contain a phonetic radical that provides some clues to character’s pronunciation, previous studies have indicated phonological awareness produced significant correlations with reading in Chinese (e.g., Ho and Bryant, 1997). Besides, children’s rapid automatized naming and IQ have a strong relationship with reading comprehension (Pammer and Kevan, 2007; Araújo et al., 2015; Weng et al., 2016). Therefore, word reading, vocabulary knowledge, rapid digit naming, IQ, and phonological awareness were included as the control variables. With these variables being controlled to minimize the bias from extraneous variables, the present research aimed to investigate the developmental relationship between morphological awareness and reading comprehension across elementary grades.

To give an overview, the aim of the present study was to examine the longitudinal relation between Chinese children’s homophone awareness, homograph awareness, compounding awareness and reading comprehension simultaneously, during the early – middle – upper elementary school years. To this end, we use a 1-year longitudinal study and three structural equation model with a sample of 439 Chinese-speaking students in Grades 1, 3, and 5, after controlling for word reading, vocabulary knowledge, IQ, rapid automatized naming, and phonological awareness measured at the initial level. Based on the literature review, the present study aimed to answer the following questions. (1) Is there bidirectional association between children’s compounding awareness, homograph awareness, homograph awareness and reading comprehension across Grades 1, 3, and 5? (2) Does compounding awareness has a stronger positive effect on reading comprehension than homograph awareness and homograph awareness across Grades 1, 3, and 5? (3) Does the unique role of morphological awareness in reading development is of increasing importance with the grade increases?

## Materials and Methods

### Participants

This study is part of an ongoing longitudinal study (e.g., Cheng et al., 2017) which was approved by the relevant Research Ethics committee of Beijing Normal University. Prior to conducting the study, written informed consent was obtained from the parents of all children participants.

The initial sample of participants were 439 children from Grades 1, 3, 5 in two urban elementary schools in the Shanxi province in China. Each age group of the participants were recruited and tested two times from Grade 1 to 2, Grade 3 to 4, and Grade 5 to 6, respectively. The testing times were in the spring [Time 1 (T1); *n* = 143] of Grade 1, and spring [Time 2 (T2); *n* = 140] of Grade 2; the fall [Time 1 (T1); *n* = 141] of Grade 3, and fall [Time 2 (T2); *n* = 136] of Grade 4; and the fall [Time 1 (T1); *n* = 145; 73 girls] of Grade 5, and fall [Time 2 (T2); *n* = 144; 71 girls] of Grade 6. Their mean ages for Grades 1, 3, and 5 at first testing were 6.81, 8.30, and 10.38 years old, respectively. None of the children had a known cognitive or developmental disability. All students were native Chinese speakers. From the original sample at T1 across the three Grades, 3 (2.10% attrition), 5 (3.54% attrition), 1 (0.69% attrition) children were eliminated because of missing values on the assessments at T2 in Grades 1, 2 and 3, respectively. Attrition at any time point in this study was found to be mainly due to moving out of the school district.

### Measures and Procedure

A battery of measures, including word reading, vocabulary knowledge, IQ, phonological awareness, rapid digit naming, homophone awareness, homograph awareness, compounding awareness, and reading comprehension, were administered. Except for the group test of reading comprehension, all other measures were administered individually. The order of test administration was random across classrooms. The morphological awareness and reading comprehension tests were administered to the children at the beginning of November in the fall semester(T1), and the same time the next year (T2), except the first Graders. Given the limited level of first-Graders’ reading development, the initial test of these students was implemented at the beginning of April in the spring semester, and the same time the next year (T2). In addition, control variables, including word reading, vocabulary knowledge, IQ, rapid automatized naming, and phonological awareness, were assessed at T1 only. To reduce fatigue, data collection at each time point was completed over 1 week. All measures were administered by trained experimenters who were graduate students majoring in psychology or education. The testing of vocabulary knowledge, IQ, homophone awareness, homograph awareness, compounding awareness, and reading comprehension, lasted for approximately 20–35 min, and the other two lasted for approximately 5 to 10 min.

#### Homophone Awareness

Following Liu and McBride-Chang (2010), the morphological homophone awareness task consisting of 2 practice and 12 test items, was administered to assess children’s homophone awareness. Children were first orally presented with a target morpheme in a two-character word, and then asked to name another word with this same morpheme to confirm that they knew the target morpheme and its pronunciation. For example, the target morpheme (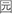/yuan2/) was orally presented together with a sample word containing it (e.g., 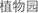/zhi2– wu4– yuan2/ “botanical garden”). The children were then asked to construct new words that included the same morpheme (e.g., 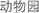/dong4-wu4-yuan2/ “zoo”) and then to produce as many homophones (words containing the same syllable /yuan2/) as possible (e.g., 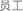/yuan2– gong1/ “employee” or 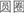/yuan2– quan1/ “ring”). The scores were based on the correct number of different homophone morphemes produced, each correct word with different homophone morphemes being given 1 point. The scores were obtained based on the number of different homophone morphemes produced. Thus, there was no maximum score.

#### Homographic Awareness

Following Shu et al. (2006), we developed a homographic awareness task administered orally, which consisted of two practice and 12 test items. In this task, children were first presented with a two-syllable Chinese word (e.g., 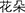/hua1– duo3/ “flower”) that included the target morpheme 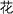/hua1/. Children were asked to produce two words with the target morpheme. One word was supposed to contain the same meaning as the target morpheme, such 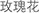/mei2 – gui4 – hua1/ “rose.” The other word was supposed to have a different meaning from that of the target morpheme but the same pronunciation and written form, such as 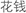/*hua1*– *qian2/* “spend”. The word with same homograph morpheme and different homograph morpheme being given 1 point each.

#### Compounding Awareness

This task tested children’s ability to manipulate the morphological structure of compound words. Similar to Liu and McBride-Chang’s study (2010), students were presented orally a question/scenario and then asked come up with a novel word that fits the scenario (Cheng et al., 2017). This task included two parts. The first part (12 of items) involved manipulating two morphemes, whereas the second part (8 of items) involved three morphemes. For example, one question in first part was, “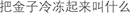 (What should we call a bird like a frog?)”. The best answer was 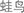 (/wa1– niao3/, frog bird). Another question in second part was, “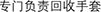 (What do we call a factory that is specially used to recycle glove ?)”. The best answer was 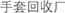 (/shou3– tao4– hui2– shou1– chang3/, glove-recycling-factory). Two independent scorers rated the same responses. Discrepancies in ratings were resolved. Following the scoring scheme of Liu and McBride-Chang’s study, a scale of 0–3 was used to score the responses based on two criteria: conciseness and correctness of word structure. This test included 8 practice items and 20 test items. If the children did not answer five consecutive items, the test was stopped.

#### Reading Comprehension

The participants in each grade read one age-appropriate passage and answered multiple-choice or open-ended questions. For first grade students, one narrative passage and 18 multiple choice questions were chosen from a Chinese reading ability scale (Mo, 2004), Prince Nezha Conquers the Dragon King. The test was age-appropriate for early grade Chinese students and had been used in previous studies with good reliability and validity (Cheng et al., 2016). The test for Grade 3 included a passage entitled An Unbelievable Night (Wen, 2005), 1 sequencing question, 6 multiple-choice questions, and 5 open-ended questions. The test for Grade 5 included the passage titled Searching for Food (Wen, 2005), 1 sequencing question, 7 multiple-choice questions, and 7 open-ended questions. Each sequencing question and multiple-choice question was worth one point. Constructed response questions were scored on a 1–3 point scale in terms of the completeness and accuracy in describing the relationship of key points. Two raters independently scored all constructed-response items. The maximum score for Grades 3 and 5 students was 16 and 17, respectively.

#### Word Reading

Chinese character recognition task consisted of 150 single characters with increasing difficulty (Li et al., 2012). Children were asked to read from the beginning and stop when they failed to read 15 consecutive items.

#### Vocabulary Knowledge

Following Song et al. (2015), the vocabulary definition task was administered to assess children’s vocabulary knowledge. Children were asked to produce an explanation of the words that were orally presented (e.g., 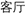/*ke4—ting1/* “parlor,” to which the best answer would be “the place to meet guests”). There were 1 practice and 32 test items. Children’s answers were rated on a scale of 0–2 by two trained psychology students based on the rating criteria. Testing stopped if a child failed on five consecutive items.

#### Rapid Automatized Naming

A rapid digit naming task used in previous research (e.g., Shu et al., 2006) was used in this study. In this task, 5 digits, specifically 1, 3, 4, 5, and 8, were repeated 5 times in a random order on a single sheet of paper. The children were instructed to say aloud the digits from left to right and from top to bottom as quickly and accurately as possible. Each child named the digits twice. The average latency across the two trials was computed to the nearest 1/100s, and errors were recorded.

#### IQ

Raven’s Standard Progressive Matrices was used to assess students’ non-verbal IQ (Raven et al., 1996). In this 60-item task, a pattern with a missing part was presented, and the children were asked to choose the appropriate one from the options to complete the target pattern. The raw IQ score was analyzed as a control variable.

#### Phonological Awareness

The children’s phonological awareness was measured by a phoneme deletion task. In the task, children were asked to produce a new syllable by deleting the target phoneme from a monosyllabic Chinese syllable. For example, children were asked to remove the “*zh”* from the syllable “*zhá*,” and the answer was “*á*”. A correct answer was given 1 point, and 0 point was given for an incorrect response or no response. There were four practice items and 12 test items, including four initial, middle and last phoneme deletion items.

### Analysis Strategy

Descriptive analyses and Pearson correlations were calculated to examine the associations among IQ, phonological awareness, rapid digit naming, word reading, vocabulary knowledge, homophone awareness, homograph awareness, compounding awareness, and reading comprehension in each Grade at T1 and T2, respectively.

To assess the bidirectional relations between components of morphological awareness (homophone awareness, homograph awareness, compounding awareness), and reading comprehension, during the early – mid – upper elementary school years respectively, a structural equation modelling (SEM) was employed in a cross-lagged model. In this model, we controlled for word reading, vocabulary knowledge, IQ, rapid digit naming, and phonological awareness, when analyzing the relationship between the three component of morphological awareness. In order to make model comparisons available, Z scores were used for children’s reading comprehension score in three grades, because different passages were used for measuring reading comprehension skills in different age groups. Statistical analyses were conducted using *Mplus* 6 (Muthén and Muthén, 1998–2012). Missing data were handled using maximum likelihood estimation with robust standard errors (MLR).

To evaluate the model fit, we used the chi-square test, comparative fit index (CFI), Tucker-Lewis index (TLI), root mean square error of approximation (RMSEA), and standardized root mean residual (SRMR). According to recommendations by several researchers (e.g., Hu and Bentler, 1999; Kline, 2011), CFI and TLI ≥ 0.90, RMSEA ≤ 0.06, and SRMR ≤ 0.08 are considered as good fit.

Finally, to examine potential differences between three groups in the specific path, multi-group analyses were conducted. Significant differences between groups for the specific path were assessed by first freely estimating the parameter and then imposing equality constraints. The significant decrements in model fit following parameter constraints were assessed with the chi-square difference test. Additionally, fit indices including χ^2^ (*df*), CFI, RMSEA, and SRMR were used to evaluate the fit of the models.

## Results

### Descriptive Statistics and Correlations

Table 1 displays the means, standard deviations, and reliabilities (coefficient alphas) for all variables across the three Grades. The raw scores were presented for all measures, with the exception of rapid digit naming, which was calculated in seconds. Generally speaking, children performance on homophone awareness, homograph awareness, compounding awareness and reading comprehension, increased from T1 to T2, in each of the three Grades.

**Table 1:** Descriptive statistics of all variables among Grade 1, Grade 3, and Grade 5.

Measure	Time	*M* (*SD*)	Reliability	Maximum
**Grade 1**				
Reading comprehension	T1	7.73 (3.26)	0.67	18
	T2	10.99 (4.00)	0.81	18
Compounding awareness	T1	14.44 (10.99)	0.85	60
	T2	25.78 (10.51)	0.84	60
Homophone awareness	T1	8.68 (4.57)	0.87	–
	T2	13.25 (6.29)	0.85	–
Homograph awareness	T1	9.26 (3.16)	0.72	24
	T2	12.12 (2.78)	0.77	24
Non-verbal IQ	T1	28.06 (9.31)	0.91	60
Rapid number naming	T1	12.54 (2.79)	0.77	–
Phonological awareness	T1	8.86 (2.92)	0.88	12
Word reading	T1	45.13 (24.94)	0.99	150
Vocabulary knowledge	T1	10.74 (5.74)	0.74	64
**Grade 3**				
Reading comprehension	T1	6.71 (2.60)	0.79	16
	T2	9.33 (3.21)	0.83	16
Compounding awareness	T1	21.71 (11.64)	0.85	60
	T2	29.81 (8.91)	0.81	60
Homophone awareness	T1	12.19 (4.73)	0.85	–
	T2	19.24 (5.97)	0.86	–
Homograph awareness	T1	11.49 (3.28)	0.75	24
	T2	14.31 (3.13)	0.74	24
Non-verbal IQ	T1	40.56 (7.09)	0.92	60
Rapid number naming	T1	10.40 (2.98)	0.79	–
Phonological awareness	T1	9.19 (2.43)	0.86	12
Word reading	T1	91.76 (19.87)	0.99	150
Vocabulary knowledge	T1	17.09 (7.24)	0.78	64
**Grade 5**				
Reading comprehension	T1	9.40 (2.77)	0.81	17
	T2	11.27 (2.69)	0.82	17
Compounding awareness	T1	32.24 (10.72)	0.82	60
	T2	37.99 (9.00)	0.83	60
Homophone awareness	T1	17.49 (5.68)	0.88	–
	T2	25.37 (6.55)	0.87	–
Homograph awareness	T1	16.67 (3.40)	0.80	24
	T2	17.41 (3.27)	0.79	24
Non-verbal IQ	T1	44.65 (6.48)	0.95	60
Rapid number naming	T1	7.60 (1.80)	0.78	–
Phonological awareness	T1	10.30 (1.79)	0.89	12
Word reading	T1	117.81 (12.00)	0.97	150
Vocabulary knowledge	T1	26.97 (6.31)	0.76	64

*Represent no maximum score. They were not necessarily the maximum score due to the open-ended nature of the morphological homophone awareness task and rapid number naming task*.

Table 2 presented the Pearson correlation matrix for all variables. The correlation analysis results showed that reading comprehension is significantly correlated with homophone awareness, homograph awareness, and compounding awareness at each time point of Grades 1, 3 and 5, except for the first Grade at T1. These results provide evidence of a developmental relationship between morphological awareness and reading comprehension.

**Table 2:** Pearson correlations between all variables among Grade 1, Grade 3, and Grade 5.

	1	2	3	4	5	6	7	8	9	10	11	
**Grade 1**												
1.T1 RC	-											
2.T2 RC	0.29**	-										
3.T1 CPA	0.11	0.38**	-									
4.T2 CPA	0.14	0.48**	0.62**	-								
5.T1 HPA	-0.01	0.31**	0.42**	0.44**	-							
6.T2 HPA	0.12	0.45**	0.39**	0.51**	0.58**	-						
7.T1 HGA	-0.02	0.36**	0.38**	0.39**	0.42**	0.36**	-					
8.T2 HGA	0.05	0.35**	0.38**	0.54**	0.38**	0.55**	0.46**	-				
9.T1 IQ	0.24**	0.39**	0.31**	0.28*	0.27**	0.21*	0.36**	0.25**	-			
10.T1RAN	-0.11	-0.18*	-0.10	-0.26**	-0.20*	-0.15	-0.11	-0.18*	-0.14	-		
11.T1PA	-0.03	0.10	0.24**	0.29**	0.17*	0.26**	0.29**	0.24**	0.08	-0.17**	-	
12.T1WR	0.40**	0.47**	0.45**	0.42**	0.34**	0.42**	0.30**	0.33**	0.30**	-0.27**	0.34**	-
13.T1VK	0.14	0.43**	0.52**	0.49**	0.48**	0.45**	0.59**	0.36**	0.37**	-0.25**	0.27**	0.34**
**Grade 3**												
1.T1 RC	-											
2.T2 RC	0.55**	-										
3.T1 CPA	0.32**	0.38**	-									
4.T2 CPA	0.47**	0.48**	0.53**	-								
5.T1 HPA	0.39**	0.21*	0.39**	0.36**	-							
6.T2 HPA	0.40**	0.42**	0.28**	0.41**	0.40**	-						
7.T1 HGA	0.49**	0.43**	0.39**	0.40**	0.41**	0.33**	-					
8.T2 HGA	0.48**	0.43**	0.44**	0.43**	0.44**	0.31**	0.44**	-				
9.T1 IQ	0.48**	0.53**	0.31**	0.39**	0.22**	0.28**	0.36	0.24**	-			
10.T1RAN	-0.14	-0.18*	-0.05	-0.13	-0.11	-0.21*	-0.03	-0.21*	-0.08	-		
11.T1PA	0.32**	0.46**	0.20*	0.29**	0.22**	0.38**	0.43**	0.46**	0.28**	-0.23**		
12.T1WR	0.55**	0.48**	0.38**	0.43**	0.41**	0.32**	0.37**	0.49**	0.31**	-0.22**	0.38**	-
13.T1VK	0.53**	0.59**	0.48**	0.59**	0.42**	0.33**	0.58**	0.58**	0.41**	-0.20*	0.39**	0.48**
**Grade 5**												
1.T1 RC	-											
2.T2 RC	0.45**	-										
3.T1 CPA	0.26**	0.32**	-									
4.T2 CPA	0.44**	0.41**	0.51**	-								
5.T1 HPA	0.27**	0.21**	0.29**	0.24**	-							
6.T2 HPA	0.38**	0.23**	0.25**	0.38**	0.50**	-						
7.T1 HGA	0.33**	0.29**	0.36**	0.42*	0.39**	0.42**	-					
8.T2 HGA	0.30**	0.29**	0.26**	0.44**	0.22**	0.42**	0.47**	-				
9.T1 IQ	0.46**	0.32**	0.18*	0.30**	0.23**	0.28**	0.24**	0.29**	-			
10.T1RAN	-0.29**	-0.15	0.00	-0.09	-0.22**	-0.28**	-0.15	-0.08	-0.04	-		
11.T1PA	0.14	0.08	0.27**	0.21*	0.31**	0.30**	0.40**	0.29**	0.19*	-0.12		
12.T1WR	0.39**	0.37**	0.27**	0.45**	0.39**	0.24**	0.39**	0.38**	0.19*	-0.37**	0.18*	-
13.T1VK	0.36**	0.45**	0.27**	0.44**	0.38**	0.23**	0.38**	0.36**	0.20*	-0.19*	0.18*	0.51**

*RC, reading comprehension; CPA, compounding awareness; HPA, homophone awareness; HGA, homograph awareness; IQ, non-verbal IQ; RAN, rapid number naming; PA, phonological awareness; WR, word reading; VK, vocabulary knowledge; ^∗^*p* < 0.05, ^∗∗^*p* < 0.01, two- tailed*.

### Reciprocal Relations Between Morphological Awareness and Reading Comprehension

To examine the longitudinal relation between morphological awareness and reading comprehension across elementary school years, we used a 1-year longitudinal study and a structural equation model with the students in Grades 1, 3, and 5, respectively.

**Figure 1:**
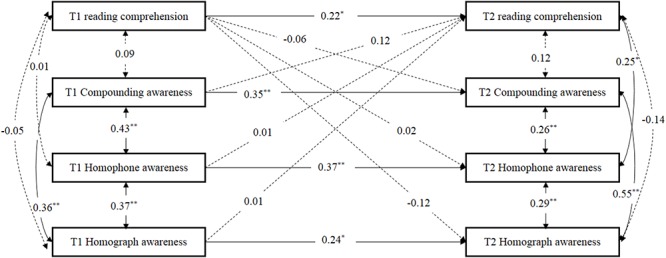
The model to evaluate the longitudinal relationships between compounding awareness, homophone awareness, homograph awareness, and reading comprehension from Grade 1 to 2, after controlling for IQ, rapid number naming, phonological awareness, word reading, and vocabulary knowledge at the initial level. T1, spring of first Grade; T2, spring of second Grade. Dashed lines indicate non-significant paths. The control variables are not shown here to simplify the representation of the model.^∗^*p* < 0.05, ^∗∗^*p* < 0.01, two- tailed.

As shown in Figure 1, the first structural equation model was used to test the bidirectional relationships between the three components of morphological awareness (compounding awareness, homophone awareness, homograph awareness), and reading comprehension over at two time points of Grade 1, after controlling for IQ, rapid number naming, phonological awareness, word reading, and vocabulary knowledge. The model showed good fit, χ^2^ (6) = 7.32 (*p* = 0.29), RMSEA = 0.04 (90% CI = 0.00–0.12), SRMR = 0.03, CFI = 0.99, TLI = 0.96. The paths for compounding awareness, homophone awareness, homophone awareness, and reading comprehension from T1 to T2 were all significant, *ps* < 0.05. After controlling for the other cognitive and linguistic covariates, the paths from compounding awareness, homophone awareness and homograph awareness at T1 to reading comprehension at T2, from reading comprehension at T1 to three components of morphological awareness at T2, respectively, were not significant.

**Figure 2:**
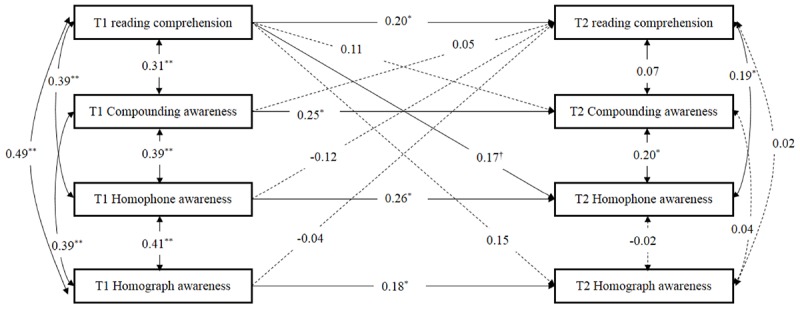
The model to evaluate the longitudinal relationships between compounding awareness, homophone awareness, homograph awareness, and reading comprehension from Grade 3 to 4, after controlling for IQ, rapid number naming, phonological awareness, word reading, and vocabulary knowledge at the initial level. T1, fall of third Grade; T2, fall of fourth Grade. Dashed lines indicate non-significant paths. The control variables are not shown here to simplify the representation of the model. †*p* < 0.08, ^∗^*p* < 0.05, ^∗∗^*p* < 0.01, two- tailed.

The second structural equation model was used to investigate the longitudinal relationship between morphological awareness and reading comprehension from Grade 3 to 4, after controlling for IQ, rapid number naming, phonological awareness, word reading and vocabulary knowledge. The most fit was satisfactory, *χ*^2^ (6) = 9.28 (*p* = 0.16), RMSEA = 0.06 (90% CI = 0.00–0.13), SRMR = 0.02, CFI = 0.99, TLI = 0.92. As displayed in Figure 2, the paths for compounding awareness, homophone awareness, homograph awareness, and reading comprehension from T1 to T2 were all significant, *ps* < 0.05. The cross-lagged path from reading comprehension at T1 to homophone awareness at T2 (standardized β = 0.17, *p* < 0.08) was marginal significance. In addition, paths from compounding awareness, homophone awareness and homograph awareness at T1 to reading comprehension at T2, from reading comprehension at T1 to compounding awareness and homograph awareness at T2, respectively, were not significant, after controlling for IQ, rapid number naming, phonological awareness, word reading and vocabulary knowledge.

**Figure 3:**
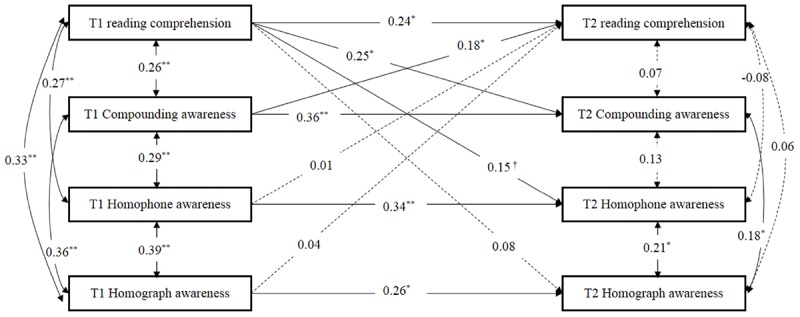
The model to evaluate the longitudinal relationships between compounding awareness, homophone awareness, homophone awareness, and reading comprehension from Grade 5 to 6, after controlling for IQ, rapid number naming, phonological awareness, word reading, and vocabulary knowledge at the initial level. T1, fall of fifth Grade; T2, fall of sixth Grade. Dashed lines indicate non-significant paths. The control variables are not shown here to simplify the representation of the model. †*p* < 0.08, ^∗^*p* < 0.05, ^∗∗^*p* < 0.01, two- tailed.

The third structural equation model for the sample from Grade 5 to Grade 6 also showed satisfactory model fit, *χ*^2^(6) = 5.26 (*p* = 0.51), RMSEA = 0.00 (90% CI = 0.00–0.09), SRMR = 0.02, CFI = 1.00, TLI = 1.02. As can be seen in Figure 3, The paths for compounding awareness, homophone awareness, homograph awareness, and reading comprehension from T1 to T2 were all significant, *ps* < 0.05. After controlling for IQ, rapid number naming, phonological awareness, word reading and vocabulary knowledge, path analyses revealed that reading comprehension had positive effect on compounding awareness from T1 to T2 (standardized β = 0.25, *p* < 0.05), and compounding awareness also had a significant and positive effect reading comprehension from T1 to T2 (standardized β = 0.18, *p* < 0.05). The cross-lagged path from reading comprehension at T1 to homophone awareness at T2 (standardized β = 0.15, *p* < 0.08) was marginal significance. In addition, the cross-lagged paths from reading comprehension at T1 to homograph awareness at T2, from homograph awareness at T1 and homophone awareness at T1 to reading comprehension at T2 were not significant. These findings indicated that only compounding awareness had a reciprocal influence on reading comprehension in the late elementary school years after controlling for word reading, vocabulary knowledge, IQ, rapid number naming and phonological awareness.

### Multiple-Group Path Analyses

To examine whether there were significant differences in the effects of compounding awareness at T1 on reading comprehension at T2 across Grades 1, 3 and 5, we first constrained the structural parameters in this path to be equal across Grades 5 and 3, which resulted in a statistically significant worsening of overall model fit (Δχ^2^ = 4.032, Δ*df* = 1, *p* < 0.05). When constraining the structural parameters in path from compounding awareness at T1 to reading comprehension at T2 across Grades 5 and 1, the fit of the constrained model was marginally significantly different from the fit of the unconstrained model (Δχ^2^ = 3.171, Δ*df* = 1, *p* = 0.07). These results indeed indicated that the association between compounding awareness at T1 and reading comprehension at T2 differed across Grades 5 and 3, and marginally significantly differed across Grades 5 and 1. Specifically, as the Figure 3 shows, compounding awareness at T1 significantly predicted and reading comprehension at T2 for high group (β = 0.18, *p* < 0.05), but not for medium group (β = 0.05, *p* > 0.05) and low group (β = 0.12, *p* > 0.05).

## Discussion

The primary goal of the present study was to investigate the longitudinal relationship between three components of morphological awareness (compounding awareness, homophone awareness, homograph awareness), and text reading comprehension among Chinese-speaking children from Grades 1 to 2, Grades 3 to 4, and Grades 5 to 6, respectively, after controlling for word reading, vocabulary knowledge, IQ, rapid number naming, and phonological awareness. The major results are as follows. (1) The initial compounding awareness predicted later reading comprehension only from Grades 5 to 6, and initial reading comprehension also made a unique contribution to later compounding awareness from Grades 5 to 6. (2) The unique role of compounding awareness in reading development changes toward a trend of increasing importance with the grade increases. (3) Reading comprehension in Grades 3 and 5 predicted homophone awareness in Grades 4 and 6 were marginal significance, respectively, but initial homophone awareness did not predict later reading comprehension across grades. (4) There no unique connection between homograph awareness and reading comprehension across grades.

The present research examined the three components of morphological awareness in the same model simultaneously to explore the unique contributions of each component to reading comprehension across elementary grades, and only identified the unique role of compounding awareness in Chinese children’s reading development and the reciprocal relationship between compounding awareness and text reading comprehension in the late elementary school years, after controlling for word reading, vocabulary knowledge and other related factors. Going beyond previous research that highlight the importance of compounding awareness for children in early grades (e.g., Liu et al., 2013; Cheng et al., 2017), the current study showed that compounding awareness is a unique predictor of Chinese reading comprehension in the late elementary school years. This finding is consistent with Kuo and Anderson (2006) notion that “morphological awareness becomes an increasingly important predictor of measures of reading as children grow older” (p. 161).

The particular importance of compounding awareness in Chinese children’s reading development may be due to the overlapping characteristics with syntactic awareness and semantic knowledge of compounding awareness (Carlisle, 1995; Shu et al., 2006; Liu et al., 2013; 2017). According to the Simple View of Reading, word decoding, and linguistic comprehension are two core skills for reading comprehension (Gough and Tunmer, 1986; Hoover and Gough, 1990). In the early stages of elementary school years, children are heavily dependent on the familiar appearance of words for decoding when learning to read (Chall, 1983; Ehri, 1995). As the grade increases, the unique role of compounding awareness in reading development changes toward a trend of increasing importance with the grade increases. The researchers noted that morphological structure of words is often conditioned by syntactic context, compounding awareness, “the understanding of how morphemes can be combined sensibly in the Chinese language” (Liu and McBride-Chang, 2010, p. 63), also shares characteristic with syntactic awareness (e.g., Li et al., 2017). In fact, manipulating morphemes as in the lexical compounding task enables children to understand “how morphemes can be combined sensibly in the Chinese language” (Liu and McBride-Chang, 2010, p. 63). Thus, even after controlling for word reading and vocabulary knowledge, children can still use the words boundary cue provided by suffixes in compounding awareness to help analyzing phrases and sentences given the absence of word boundaries, then lead to better comprehension.

There is another explanation for the unique role of compounding awareness in later reading comprehension in Chinese students from Grade 5 to 6. Children are likely to encounter many morphologically complex words in their reading, especially the morphologically complex words common in upper elementary school texts (Nagy and Anderson, 1984; Nagy et al., 1993). Children’s compounding awareness involved the ability to read key unfamiliar and complex words could then support comprehension of texts. In the present study, compounding awareness assessed by the compound production task, which taps children’ ability to manipulate morphemes and fit them into a correct structure succinctly in oral language. For example, for the question in the compound production task, “
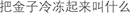
” (“What should we call the procedure in which gold is frozen?”), the best answer is 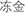 (dong4 jin1; frozen- gold). To construct this novel compound, students need to extract the critical morphemes 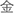 (gold) and 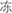 (frozen), and combine them in the correct word structure
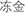
(frozen-gold). When encountering unfamiliar morphologically complex words in reading, children may extract familiar morphemes and then combine the morphemes into words, in order to infer the meaning of unknown words, thereby fostering understanding of the gist of a text. Therefore, it is unsurprising that compounding awareness is important for successful reading comprehension, after controlling for word reading and vocabulary knowledge that are familiar to children.

Contrary to our expectations, children’s homophone awareness and homograph awareness at T1 did not predict reading comprehension at T2 across grades in the present study after controlling for word reading, vocabulary knowledge and other related factors. The importance of homophone awareness and homograph awareness in reading comprehension in Chinese is well documented in literature (Tong et al., 2009; Liu et al., 2013), however, those results were not supported by the findings in this study. We conjecture that the lack of significant effect in the present study might be attributed to homophone awareness and homograph awareness only involve the ability to identify and manipulate a specific morpheme; and the specific morpheme presents in any given word that is familiar to children. Reading comprehension involves the process of meaning-making, and particular relies on accurate mental representation of the key complex and unfamiliar words in text (Deacon et al., 2014). Children are likely to encounter many morphologically complex and unfamiliar words in their reading, especially during the elementary school years (Nagy and Anderson, 1984; Nagy et al., 1993). Further, children are even more likely to encounter morphologically complex and unfamiliar words in written language than in oral language (Chafe and Danielewicz, 1987). However, homophone awareness and homograph awareness all assessed by the homophone/homographic production task in orally, demand conscious access to specific morphemes, which in the words they are familiar with. Thus we suspected that homophone awareness and homograph awareness inability to infer the meaning of key complex and unknown words couldn’t then support comprehension of texts, at least in the present study.

It is worth noting that, in the early and middle of primary school, the present study failed to detect unique role of three components of morphological awareness in text reading comprehension among Chinese-speaking children. Yeung et al. (2011) also failed to detect a unique connection between morphological awareness and reading comprehension in early stage of reading, after controlled for both word reading and vocabulary. As mentioned earlier, children are heavily dependent on the familiar appearance of words for decoding in the phase of “learning to read” (Chall, 1983; Ehri, 1995). In a study with Chinese students from grades 1 to 2, found the relationship between initial status and growth rates of compounding awareness and reading comprehension were fully mediated by word reading (Cheng et al., 2017). One meta-analysis also showed that word reading would be more important for younger readers than older readers in reading (García and Cain, 2014). As suspected by Deacon et al. (2014), who notion that “there appears to be partial or complete mediation between morphological awareness and reading comprehension by word reading in middle elementary school, with direct effects only between morphological awareness and reading comprehension by upper elementary school.” Kieffer et al. (2013) also found direct effects between morphological awareness and reading comprehension were found only in grade 6 (Kieffer and Lesaux, 2012). It can be speculated that children’s morphological awareness might initially support reading comprehension through its effects on word reading and vocabulary knowledge (Deacon et al., 2014; Cheng et al., 2017), with more direct effects on reading comprehension for older children (e.g., Perfetti et al., 2005; Li et al., 2017).

More interestingly, consistent with previous studies (Kuo and Anderson, 2006; Wu et al., 2009; Cheng et al., 2017), the present study also found that children’s reading comprehension at T1 significantly predicted compounding awareness at T2 from Grades 5 to 6; and reading comprehension in Grades 3 and 5 predicted homophone awareness in Grades 4 and 6 were marginal significance, respectively. The reciprocal relationship between children’s compounding awareness and reading comprehension for upper elementary grades suggests that compounding awareness develops with the accumulation of reading experience during later years of elementary school. Reading may help children to map morphemes onto Chinese characters, which could promote the development of an increasingly sophisticated understanding of the relationships among morpheme meanings, pronunciations, and orthographic representations. With the accumulation of reading experience, children can develop an abstract understanding of critical morphemes (homophones) and extract the structure of compounding words that they encounter in reading, thus supporting the development of morphological awareness (Kuo and Anderson, 2006; Cheng et al., 2017; Li et al., 2017). As children grow, more skilful reading experiences could promote the development of homophone awareness and compounding awareness.

The current study helps to clarify the inconsistent results about the reciprocal relationship between morphological awareness and reading comprehension in Chinese children. Cheng et al.’s longitudinal study (2015) found a reciprocal relationship between morphological awareness and reading comprehension in Chinese children from Grades 1 to 2. However, Wu et al. ’s interventional study (2009) only found such a reciprocal relationship in third grade, not in second grade. The present study included three components of morphological awareness (e.g., homograph awareness, homograph awareness, and compounding awareness), and the results showed that children’s compounding awareness and reading comprehension had a bidirectional effect only in grades 5 to 6, but not in grades 1 to 2 and in grades 3 to 4, after controlling for word reading, vocabulary knowledge, IQ, rapid number naming, and phonological awareness. Our results have extended the previous findings by exploring the bidirectional relationship between three components of morphological awareness and reading comprehension across elementary grades.

Several limitations in design and measurement should be acknowledged. First, in the current study, children completed the same tasks of morphological awareness and reading comprehension at the beginning and end of the year. Although there was one-year interval between repeated testing, future studies should develop equivalent tests for morphological awareness and reading comprehension to reduce practice and fatigue effects. Second, because standardized reading comprehension measures are not available in Chinese, the reliability and validity of the reading comprehension test need to be further examined in future research.

Despite these limitations, to our knowledge, this study is among the first to investigate three components of morphological awareness in Chinese: compounding awareness, homograph awareness and homophone awareness and the possible reciprocal relation between reading comprehension and each component of morphological awareness across elementary grades in a longitudinal design. The research clearly contributes to our understanding of the dynamic relationship between morphological awareness and reading comprehension during the early – mid – upper elementary school years. These current findings have implications for instructional practices for children’s early reading development and development of morphological awareness. More specifically, teachers can enhance the instruction of compounding awareness to facilitate students’ reading development throughout elementary school years, and emphasize on text comprehension instruction which will promote morphological awareness in the middle and late elementary school years.

## Author Contributions

RX, JZ, and XW conceived and designed the study. RX, JZ, and XW acquired, analyzed, and interpreted the data. RX, JZ, XW, and TN drafted the work and revised it critically for important intellectual content.

## Conflict of Interest Statement

The authors declare that the research was conducted in the absence of any commercial or financial relationships that could be construed as a potential conflict of interest.
